# Ultrasound-Guided Percutaneous Fasciotomy of Three Compartments of the Leg for Chronic Exertional Compartment Syndrome in a High-Performance Contemporary Dancer: A Case Report

**DOI:** 10.7759/cureus.112001

**Published:** 2026-07-03

**Authors:** Emilio Remolina Sánchez Rucobo, Ana M Serrano-Ardila, Iturbide A Ponce de Leon Sandoval, Jacobo Kerbel, Gilberto Antonio Apodaca Ramos, Nizaguie G Ramos Rocandio, Francisco G Castillo Vazquez

**Affiliations:** 1 Orthopedics and Traumatology Center, American British Cowdray (ABC) Medical Center, Mexico City, MEX

**Keywords:** athlete, cecs, chronic exertional compartment syndrome, exercise-induced leg pain, minimally invasive fasciotomy, percutaneous fasciotomy, sports medicine, ultrasound-guided fasciotomy

## Abstract

Chronic exertional compartment syndrome (CECS) is a frequently underdiagnosed cause of exercise-induced leg pain that is activity-dependent. We present the case of a 20-year-old female, high-performance contemporary dancer with an approximately three-year history of progressive leg pain refractory to an exhaustive multimodal conservative treatment including NSAIDs, neuromodulators, physiotherapy with myofascial release, and botulinum toxin infiltration of three compartments. Complementary diagnostic studies including musculoskeletal Doppler ultrasound, magnetic resonance imaging (MRI), and electrodiagnostic studies yielded negative results, consistent with the dynamic nature of the condition. Based on the pathognomonic symptom pattern, imaging evolution, exclusion of neurological and vascular etiologies, and lack of results with conservative measures, a presumptive clinical diagnosis of multi-compartment CECS was established after exclusion of alternative diagnoses. In November 2025, the patient underwent ultrasound-guided percutaneous fasciotomy of the anterior, lateral, and superficial posterior compartments of the left leg via three minimally invasive incisions under regional anesthesia and sedation. Intraoperative reduction of compartmental tension was directly observed following each fascial release. A minor wound dehiscence resolved without further intervention. Serial visual analog scale (VAS) scores demonstrated progressive pain resolution (preoperative 9/10, three weeks 4/10, six weeks 3/10, three months 2/10, and six months 2/10), with a successful return-to-dance program protocol at final follow-up returning to old performance levels. This case highlights the diagnostic challenges of CECS in athletes, the limitations of conservative measures, and the technical feasibility of a simultaneous three-compartment ultrasound-guided percutaneous fasciotomy as a safe, effective, and minimally invasive solution.

## Introduction

Chronic exertional compartment syndrome (CECS) is a recognized but frequently misdiagnosed cause of activity-related lower extremity pain. The condition arises from a reversible, exercise-induced increase in intracompartmental pressure (ICP) within the non-compliant osseofascial compartments of the leg, leading to transient muscular ischemia, pain, and functional limitation that resolves with rest [1,2]. CECS primarily affects young, physically active individuals engaged in repetitive high-impact activities such as distance runners, military recruits, field athletes, and dancers [1,3]. The anterior compartment is the most frequently involved, although multi-compartment syndrome is reported to be present in up to 75% of surgical cases [2].

Diagnosis of CECS represents a significant clinical challenge. Complementary diagnostic studies including musculoskeletal ultrasound, conventional MRI, and electrodiagnostic studies often yield normal findings due to the dynamic increase in ICP, which is activity-dependent [1,2]. Direct intracompartmental pressure measurement before and after exercise, using the Pedowitz criteria (pre-exercise ICP ≥15 mmHg, one-minute post-exercise ≥30 mmHg, and/or five-minute post-exercise ≥20 mmHg), has historically been considered the diagnostic gold standard [3]. However, its invasive nature, inter-operator variability, and documented false-negative rate of up to 15% on single-session testing limit its reliability [4-6]. The absence of a universally accepted, non-invasive diagnostic standard contributes to diagnostic delays that commonly exceed two years from symptom onset to definitive diagnosis [1].

Conservative management like activity modification, physiotherapy, and botulinum toxin type A injection may offer temporary relief in selected patients; however, the underlying structural physiopathological cause cannot be fully resolved without surgical decompression [7-9]. Fasciotomy remains the definitive treatment for refractory CECS, with symptom relief rates exceeding 80% in most published series [10,11]. Ultrasound-guided percutaneous techniques have increasingly gained acceptance as minimally invasive alternatives to open fasciotomy, offering comparable decompression with reduced soft tissue trauma, lower wound complication rates, and earlier return to sport [12].

Although CECS is well recognized in athletes, published data specifically addressing its presentation and surgical management in contemporary dancers and performing-arts athletes remain scarce. To our knowledge, no previous case report has described simultaneous ultrasound-guided percutaneous fasciotomy of the anterior, lateral, and superficial posterior compartments in a contemporary dancer. We present this case to illustrate the diagnostic challenges common to CECS, the limitations of conservative treatment, and the feasibility and effectiveness of a three-compartment percutaneous ultrasound-guided fasciotomy approach.

## Case presentation

Clinical history

A 20-year-old female with no significant past medical or surgical history presented with an approximately three-year history of progressive exertional pain in the left leg. She was a high-performance contemporary dancer training five to six hours daily at a competitive level. The patient described a burning, heavy sensation predominantly affecting the anterior and posterior region of her left leg, with a peak visual analog scale (VAS) score of 9/10 during high-intensity activity. Symptoms developed consistently following dance training and resolved partially with rest. She reported paradoxical worsening of symptoms with the use of compression bandages and noted exacerbation during air travel. There was no history of tibial trauma, stress fracture, peripheral vascular disease, or neurological complaints at rest. Physical examination at rest revealed tenderness to palpation over the anterior and posterior compartments of the left leg, with no signs of neurovascular compromise and preserved range of motion of the ankle and knee.

Diagnostic workup and clinical timeline

The diagnostic evolution is summarized in Table 1. During an acute pain episode in March 2022, a musculoskeletal Doppler ultrasound performed in the emergency department demonstrated no pathological findings. A 3T MRI of the left leg in October 2022 demonstrated diffuse periosteal edema along the anterior tibial diaphysis leading to an initial diagnosis of tibial periostitis, a non-specific symptom [6]. Despite an intensive conservative treatment including NSAIDs, analgesics, neuromodulators (pregabalin), conventional rehabilitation, and compression bandaging, symptoms persisted. A follow-up 3T MRI in March 2023 demonstrated progression to a muscular edema pattern, with involvement of the medial gastrocnemius, popliteus, and lateral gastrocnemius, accompanied by scarce perifascial fluid. The deep posterior and anterior compartment muscles showed no signal abnormality. Based on this evolution, the diagnosis of CECS was clinically established by the treating surgeon in March 2023. In June 2023, the patient was hospitalized for an acute pain exacerbation. Following inpatient analgesia, she received botulinum toxin type A infiltration of the anterior, lateral, and posterior compartments. Although initial response was partial improvement, her symptoms recurred. 

**Table 1:** Clinical timeline VAS: Visual analog scale, US: Ultrasonography, NSAID: Non-steroidal anti-inflammatory drug, EBL: Estimated blood loss.

Date	Event	Action/Outcome
March 2022	First emergency consultation; VAS 9/10.	Musculoskeletal Doppler US: no pathological findings.
October 2022	3T MRI left leg.	Periosteal edema tibial diaphysis; impression: periostitis/stress fracture — initial misdiagnosis.
Oct 2022-Mar 2023	Extensive conservative management.	NSAIDs, analgesics, neuromodulators, rehabilitation, compression bandages.
March 2023	Follow-up 3T MRI left leg.	Muscular edema: gastrocnemius medial, popliteus, gastrocnemius lateral; perifascial fluid. Clinical CECS diagnosis established.
June 2023	Hospitalization for acute pain exacerbation.	Inpatient analgesia; botulinum toxin type A infiltration (anterior, lateral, posterior compartments) at external centre; transient partial relief.
Dec 2023	Baropodometry assessment.	Compensatory gait patterns; abnormal plantar pressure distribution during stance phase.
October 2025	Soft-tissue ultrasound, left leg.	Morphological changes in soleus; bilateral slow tibial venous flow, predominantly left; normal arterial caliber.
October 2025	EMG and nerve conduction study, bilateral lower extremities.	Normal; no denervation or myopathy at rest — nerve entrapment excluded.
10 Nov 2025	Ultrasound-guided percutaneous three-compartment fasciotomy.	Surgical time 90 min; EBL 20 mL; direct intraoperative compartmental decompression confirmed; no complications.
11 Nov 2025	Discharge.	Neurovascular status intact; Jones bandage removed.
2 weeks	Minor wound dehiscence at posteromedial incision.	Conservative wound care; healed by secondary intention; no infection or reoperation.
3 weeks	Outpatient follow-up	VAS 4/10 on exertion; burning pain at rest resolved
6 weeks	Outpatient follow-up	VAS 3/10; normalized muscle tone; progressive return-to-dance programme initiated
3 months	Outpatient follow-up	VAS 2/10; near complete resolution of exertional pain; return to dance
6 months	Final follow-up	VAS 2/10; near complete resolution of exertional pain; return to competitive level.

A baropodometric assessment in December 2023 documented compensatory gait patterns and abnormal plantar pressure distribution during stance. A soft-tissue ultrasound of the left leg in October 2025 demonstrated morphological changes in the soleus muscle with hypoechoic zones and increased volume, along with bilateral slow tibial venous flow of predominantly left-sided predominance; normal arterial caliber was confirmed, effectively excluding arterial etiologies. Electromyography and nerve conduction studies performed bilaterally in October 2025 were normal, with no evidence of denervation or myopathy at rest, excluding peripheral nerve entrapment syndromes [1,2].

Based on the pathognomonic symptom pattern, progressive dynamic imaging changes, systematic exclusion of alternative diagnoses, and exhaustion of conservative measures, a clinical suspicion of multi-compartment CECS of the left leg was established. Formal intracompartmental pressure measurement was not performed; the diagnosis was supported intraoperatively by immediate muscular expansion following fascial release and later by postoperative clinical improvement [3,4].

Surgical technique

**Figure 1:**
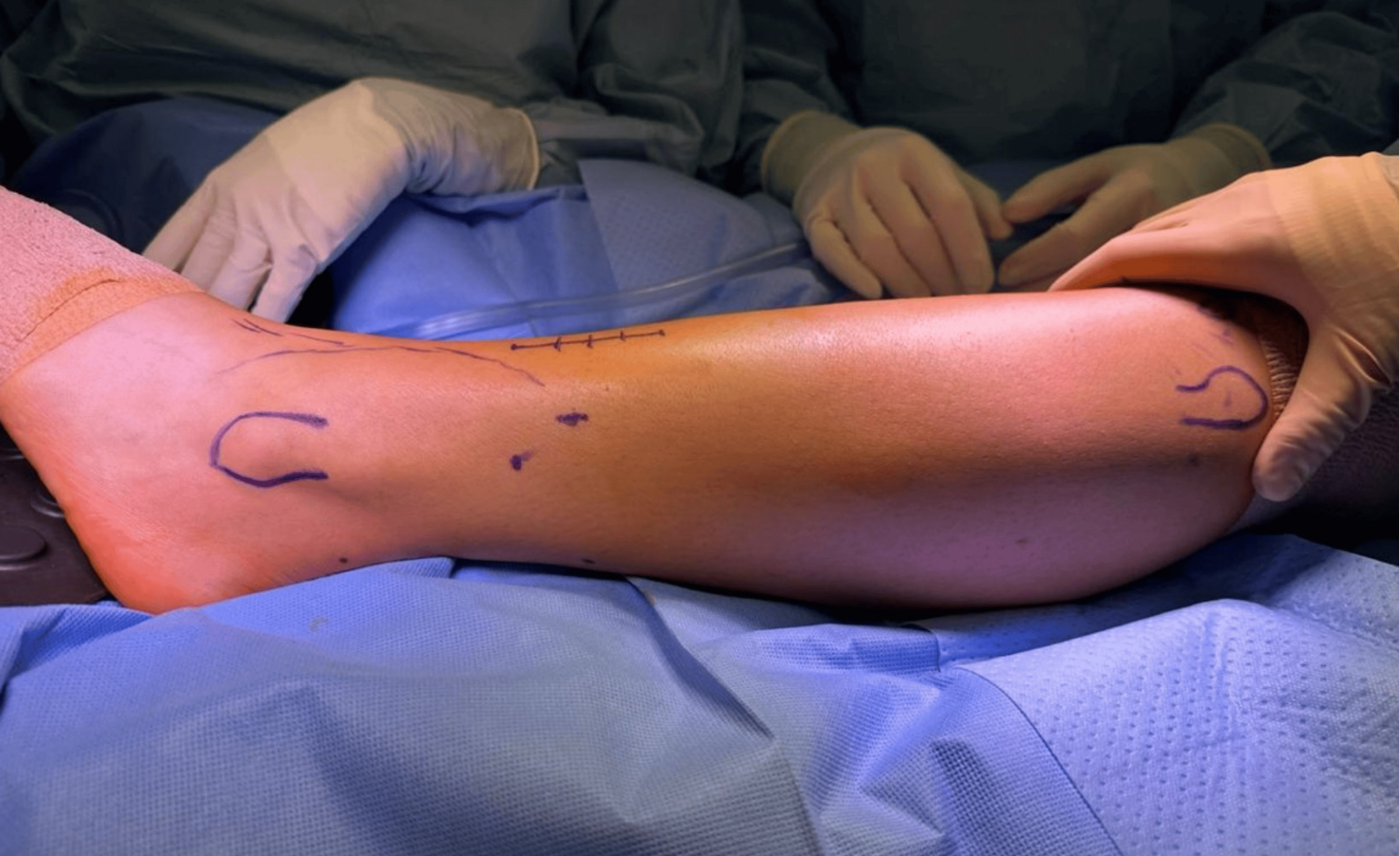
Preoperative surgical marking of the left leg The lateral incision (approximately 6 cm) is marked between the tibial spine and the anterior border of the fibular diaphysis, 10 cm proximal to the lateral malleolus, for simultaneous decompression of the anterior and lateral compartments. The posteromedial incision site (approximately 3 cm) is marked medial to the osseous border of the tibia. Anatomical landmarks including the fibular head and lateral malleolus are highlighted with a surgical marker.

In November 2025, the patient underwent elective ultrasound-guided percutaneous fasciotomy under regional anesthesia and sedation. No tourniquet or exsanguination techniques were used during the procedure. Preoperative anatomical landmarks were identified and marked on the skin prior to antiseptic preparation (Figure 1). The patient was positioned supine with a bump beneath the left hip to provide slight internal rotation and improve lateral compartment access.

Three incisions were used to access all three compartments: a lateral incision of approximately 6 cm between the tibial spine and the anterior fibular border, 10 cm proximal to the lateral malleolus, for the anterior and lateral compartments; a 4 cm lateral incision for distal decompression and a posteromedial incision of approximately 3 cm, 2 cm medial to the tibial border for the superficial posterior compartment.

**Figure 2:**
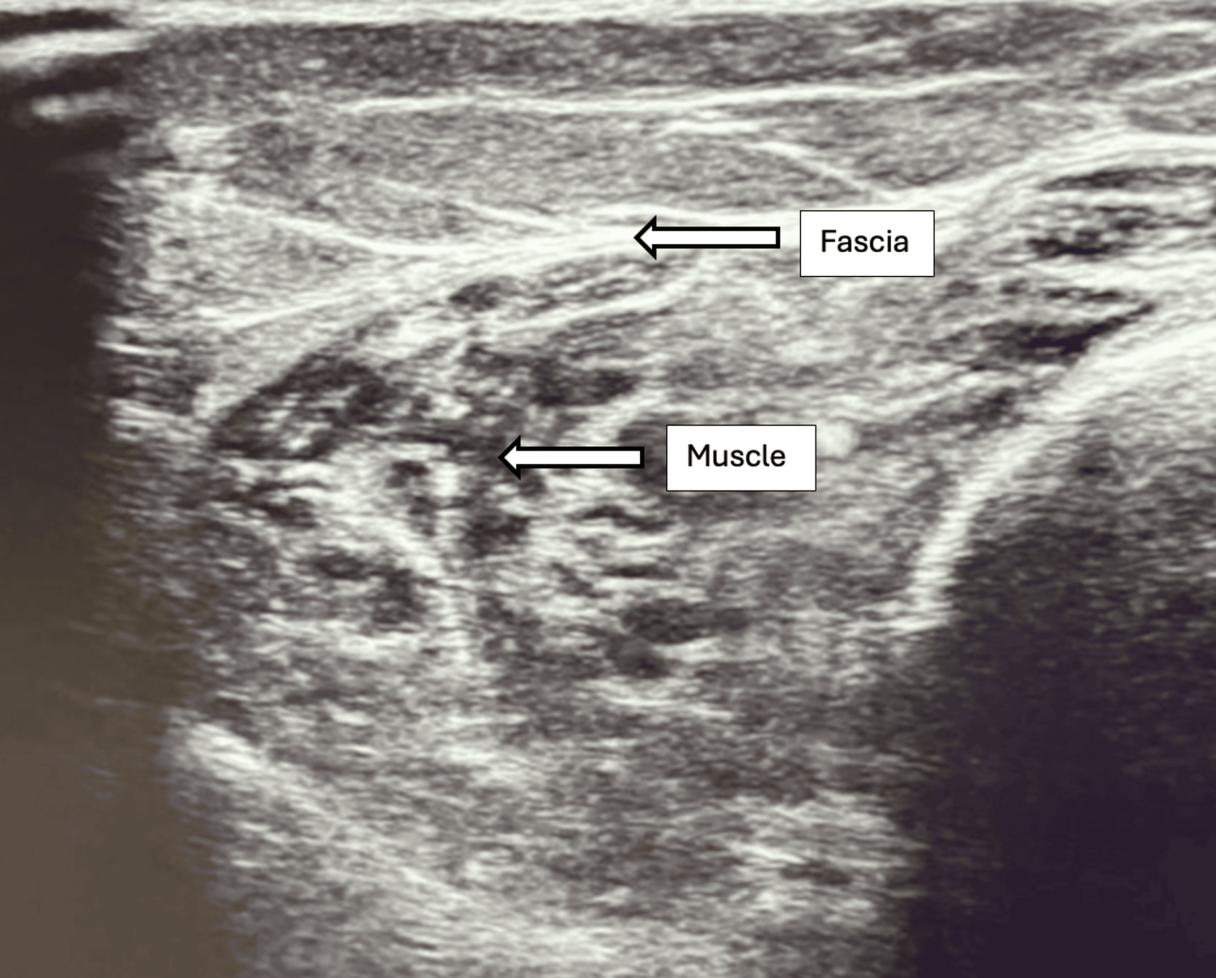
Intraoperative real-time ultrasound image during percutaneous fasciotomy of the anterior compartment Sonographic guidance was used throughout the procedure to identify the compartmental fascia, perform hydrodissection to separate fascia from the underlying muscle, and continuously monitor the position of the hook blade relative to the superficial peroneal nerve, which was identified and protected prior to fascial incision.

**Figure 3:**
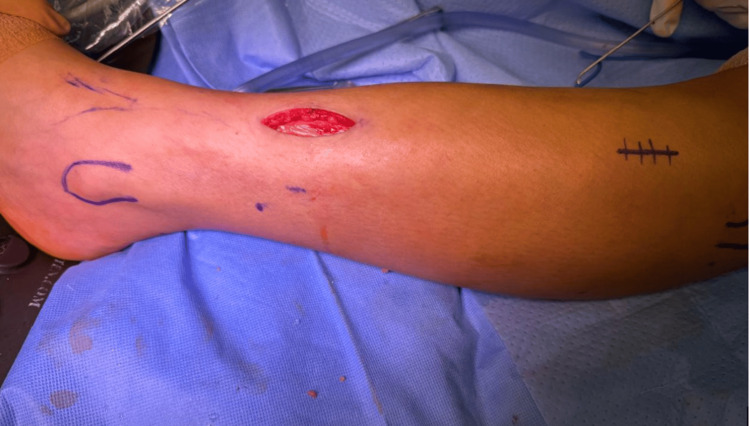
Intraoperative photograph of the lateral incision following anterior compartment decompression The minimally invasive incision provides access to both the anterior and lateral compartments. Muscular expansion through the fascial defect is directly visible following fasciotomy, confirming adequate compartmental release.

Via the lateral incisions, real-time linear ultrasound was used to identify the compartmental fascia and locate the superficial peroneal nerve (SPN) prior to any fascial intervention. The SPN emerges from the deep fascia on average 116 mm proximal to the lateral malleolus [13,14]. Hydrodissection with saline solution was performed between the fascia and the underlying muscle to create a safe dissection plane (Figure 2). A fasciotomy blade was advanced under continuous sonographic visualization to release the anterior compartment fascia longitudinally. Direct visualization of muscular expansion and reduction of compartmental tension confirmed adequate decompression (Figure 3). The lateral compartment fascia overlying the peroneus brevis was subsequently released through the distal incision.

Via the posteromedial incision, the saphenous nerve and great saphenous vein were identified and protected under ultrasound guidance prior to fasciotomy. The superficial posterior compartment fascia was incised longitudinally with confirmed direct decompression. All three compartments demonstrated immediate visible muscle expansion and palpable reduction of fascial tension. Wound closure was performed with Monocryl 3/0 inverted sutures (deep layer) and subcutaneous Monocryl 4/0 (superficial layer), reinforced with Dermabond Prineo tissue adhesive. A Jones compression bandage was applied for 24 hours. Total operative time was 90 minutes; estimated blood loss was 20 mL.

Postoperative course and follow-up

**Figure 4:**
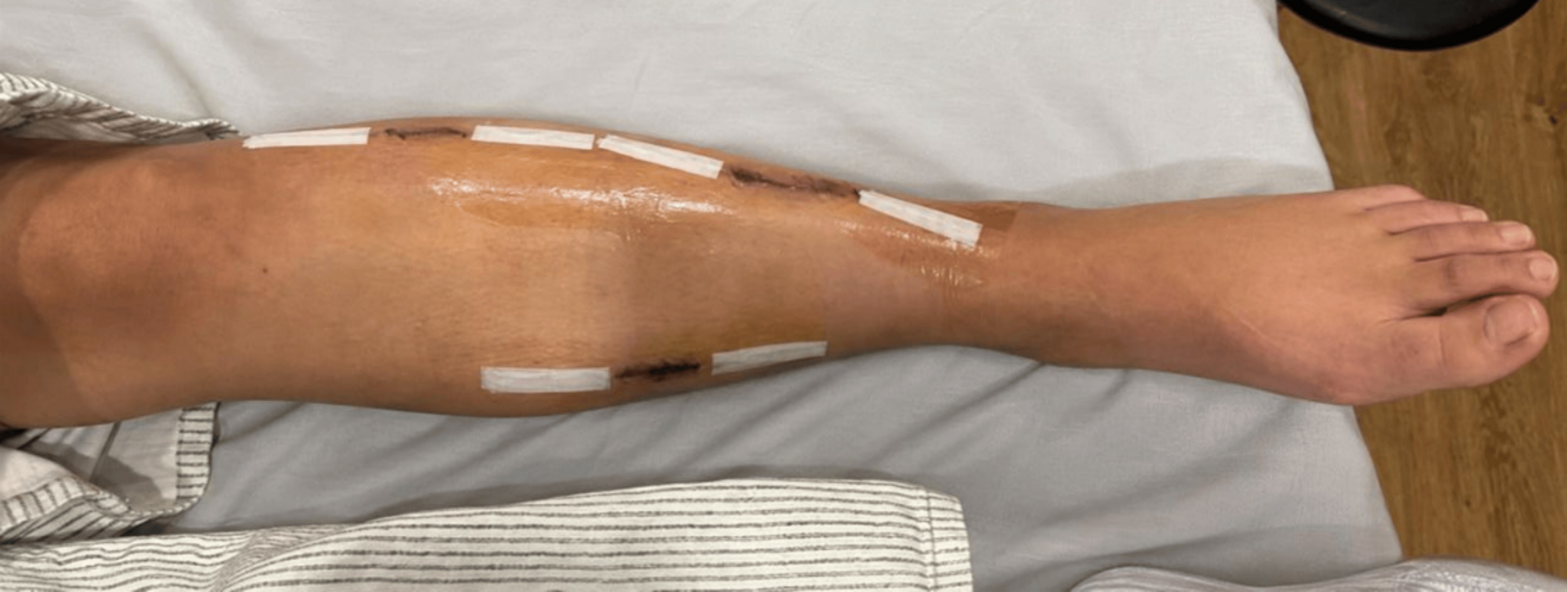
Postoperative appearance of the left leg at two-week follow-up The lateral incisions and posteromedial incision are visible, reinforced with adhesive strips over tissue adhesive (Dermabond Prineo). Both wounds demonstrate satisfactory healing progression. The minor dehiscence at the posteromedial incision site resolved by secondary intention without antibiotic therapy or wound revision.

The patient was discharged on the first postoperative day with intact distal neurovascular status and voluntary movement of all fingers and hallux. Perioperative antibiotic prophylaxis with cephalothin was administered. During the second postoperative week, ecchymosis and a minor wound dehiscence at the posteromedial incision were noted (Figure 4). This was managed conservatively and healed by secondary intention without wound revision or antibiotic therapy.

Serial VAS pain scores are presented in Table 2. The primary burning pain resolved following surgery. VAS at three weeks was 4/10, representing a 55.6 % reduction from the preoperative score of 9/10. At six weeks, VAS was 3/10, with normalized muscle tone and symmetric limb volumes; a progressive return-to-dance program was initiated under supervised rehabilitation. At three-month and six-month follow-up, VAS was 2/10, with near-complete resolution of exertional compartmental pain and successful return to dance training.

**Table 2:** Visual analog scale (VAS) pain scores during follow-up

Time Point	VAS (0–10)	Clinical Status
Preoperative	9/10	Constant exertional burning/heaviness; unable to complete full training sessions
3 weeks postoperative	4/10	Burning pain resolved; residual mild exertional discomfort; ambulating normally
6 weeks postoperative	3/10	Normalized muscle tone; progressive return-to-dance program initiated
3 months postoperative	2/10	Near complete resolution of exertional pain; no symptom recurrence
6 months postoperative	2/10	Near complete resolution of exertional pain; no symptom recurrence

## Discussion

This case illustrates three relevant points of practical importance to orthopaedic surgeons and sports medicine practitioners managing exertional leg pain in high-performance athletes. The first teaching point concerns the diagnostic challenge intrinsic to CECS. Complementary diagnostic studies are expected to be normal and therefore cannot exclude the diagnosis when negative [1,2]. In this case, the initial MRI finding of periosteal edema along the tibial diaphysis prompted a diagnosis of tibial periostitis, delaying recognition of the compartmental pathology for approximately one year. This early MRI pattern represents a non-specific finding that may reflect periosteal stress reaction rather than primary compartmental pathology, and its misinterpretation as periostitis is a recognized diagnostic pitfall in CECS. The serial MRI evolution from periosteal to muscular involvement with perifascial fluid between October 2022 and March 2023 reflects the progressive muscular stress characteristic of multi-compartment CECS, which helped with the diagnosis [6]. The normal bilateral EMG and nerve conduction studies, demonstrating absence of denervation or myopathy at rest, appropriately excluded peripheral nerve entrapment syndromes, consistent with the established principle that ischemic neurophysiological changes in CECS occur transiently during exercise and produce no permanent electrophysiological findings at rest [1]. Similarly, the absence of pathological findings on resting musculoskeletal Doppler ultrasound during the initial emergency consultation is expected in CECS, as intracompartmental pressure elevation and microvascular compromise occur dynamically during exertion and normalize with rest, yielding a false negative result under resting conditions. Post-exercise dynamic MRI and near-infrared spectroscopy are emerging as the most promising non-invasive diagnostic modalities for CECS [6], though neither was available in the institutional context in which this patient was managed.

The second point concerns the fundamental limitation of conservative management. Botulinum toxin type A has been proposed as a temporary measure in CECS based on its muscle-volume reducing effect, which mechanically decreases peak ICP during exercise [9]. Published series have reported initial clinical response rates as high as 95% [9]; however, recurrence rates approaching 57% at a median five-month follow-up have been documented [9]. The symptomatic recurrence observed in the present case following botulinum toxin infiltration is consistent with this reported pattern. In professional dancers, the resultant transient muscular weakness can have a negative effect, as dance technique demands maximal neuromuscular control and force. This professional incompatibility is not merely a quality-of-life consideration; the transient muscle weakness induced by botulinum toxin renders elite dancers temporarily unable to meet the neuromuscular demands of high-performance training, effectively constituting a secondary disability during the treatment period. This fundamental incompatibility between the therapeutic mechanism of botulinum toxin and the professional demands of elite dancers explains, at least in part, the failure of this intervention in the present case and highlights the importance of definitive surgical decompression without unnecessary delay in professional athletes who are unable to reduce training loads [5,10,11]. The paradoxical worsening of symptoms observed with compression bandaging in this patient further supports the mechanical nature of the syndrome, as external compression superimposed on an already constrained compartment predictably exacerbates intracompartmental pressure elevation.

Finally, it is important to highlight the technical feasibility and clinical effectiveness of simultaneous three-compartment ultrasound-guided percutaneous fasciotomy. The technique described here extends the ultrasound-guided percutaneous approach of Machado et al. [12] validated for the anterior and lateral compartments [13]. In this case, we decided to include the superficial posterior compartment via a separate posteromedial incision. The use of two discrete incisions mirrors the dual-incision cadaveric technique validated by Grechenig et al., which demonstrated successful compartmental decompression in 100% of 40 specimens with low risk of iatrogenic SPN injury when the nerve is identified and protected prior to fasciotomy [14]. Real-time sonographic guidance provided continuous visualization of the compartmental fascia, the blade, the SPN in the lateral corridor and the saphenous nerve in the posteromedial corridor, mitigating the risk of iatrogenic neurovascular injury that has been reported in minimally invasive fasciotomy performed without direct nerve identification [14-16]. The baropodometric documentation of compensatory gait patterns and abnormal plantar pressure distribution during stance phase, while non-diagnostic for CECS in isolation, provides objective evidence of functional adaptation to chronic exertional pain and may serve as an additional parameter to monitor functional recovery following surgical decompression [15]. The postoperative VAS evolution compares favourably with published benchmarks for minimally invasive fasciotomy [10,11], including the only indexed series to specifically include performing-arts athletes, which reported a 94% rate of return to pre-injury sport activity at a median of 13 weeks [13]. The single minor wound dehiscence is consistent with reported wound complication rates of 3-11% across fasciotomy techniques and resolved without further complications [10].

Several limitations of this report must be acknowledged. The six-month follow-up is relatively short; CECS recurrence is known to manifest at six to 24 months, and definitive conclusions regarding durability require extended observation. Formal intracompartmental pressure measurement was not performed pre-operatively; the diagnosis was supported by a characteristic symptom pattern, dynamic imaging evolution, systematic exclusion of alternative diagnoses, and intraoperative confirmation, the latter defined as direct visualization of immediate muscular expansion and palpable reduction of fascial tension following fasciotomy, but the absence of objective ICP data is a methodological limitation. The absence of validated patient-reported outcome measures (Tegner activity scale, EQ-5D, VISA score) at predefined time points further limits quantitative comparability with published series. Published literature specific to CECS in contemporary dancers is scarce in indexed literature [7,13], obligating extrapolation from heterogeneous athletic cohorts.

## Conclusions

CECS should be a primary diagnostic consideration in high-performance contemporary dancers presenting with refractory exertional leg pain, even when resting imaging and electrophysiological studies are normal. The diagnosis relies on clinical pattern recognition: characteristically exertional onset, prompt relief with rest, and failure of conservative management. Systematic exclusion of neurological and vascular diagnoses needs to be considered. Conservative treatment, including botulinum toxin, is limited by high recurrence rates and is incompatible with the professional demands of elite dance performance, as it can cause temporary muscle weakness. Ultrasound-guided percutaneous fasciotomy via minimally invasive incisions provides definitive decompression of three compartments simultaneously, with direct intraoperative confirmation of release, acceptable complication rates, and a favourable short-term outcome profile. Prospective comparative studies incorporating objective outcome measures and extended follow-up are required before widespread adoption.
